# Understanding Macrophage Interaction with Antimony-Doped Tin Oxide Plasmonic Nanoparticles

**DOI:** 10.3390/cells13171468

**Published:** 2024-09-01

**Authors:** Olexiy Balitskii, Viktoriya Ivasiv, Maria Porteiro-Figueiras, Phattadon Yajan, Mira Witzig, Aura Maria Moreno-Echeverri, José Muñetón Díaz, Barbara Rothen-Rutishauser, Alke Petri-Fink, Sandeep Keshavan

**Affiliations:** 1 Adolphe Merkle Institute, University of Fribourg, Chemin des Verdiers 4, 1700 Fribourg, Switzerland; viktoriya.ivasiv@unifr.ch (V.I.); maria.porteirofigueiras@unifr.ch (M.P.-F.); phattadon.yajan@unifr.ch (P.Y.); mira.witzig@unifr.ch (M.W.); auramaria.morenoecheverri@unifr.ch (A.M.M.-E.); barbara.rothen@unifr.ch (B.R.-R.); alke.fink@unifr.ch (A.P.-F.); 2 Department of Chemistry, University of Waterloo, 200 University Avenue West, Waterloo, ON N2L 3G1, Canada; 3 CQUM-Centre of Chemistry, Chemistry Department, University of Minho, R. da Universidade, 4710-057 Braga, Portugal; 4 Department of Physics, University of Fribourg, 1700 Fribourg, Switzerland; jose.munetondiaz@unifr.ch; 5 Department of Chemistry, University of Fribourg, Chemin du Musée 9, 1700 Fribourg, Switzerland

**Keywords:** antimony-doped tin oxide nanoparticles, photothermal effects, macrophages, cellular uptake, near-infrared radiation

## Abstract

Antimony-doped tin oxide nanoparticles (ATO NPs) have emerged as a promising tool in biomedical applications, namely robust photothermal effects upon near-infrared (NIR) light exposure, enabling controlled thermal dynamics to induce spatial cell death. This study investigated the interplay between ATO NPs and macrophages, understanding cellular uptake and cytokine release. ATO NPs demonstrated biocompatibility with no impact on macrophage viability and cytokine secretion. These findings highlight the potential of ATO NPs for inducing targeted cell death in cancer treatments, leveraging their feasibility, unique NIR properties, and safe interactions with immune cells. ATO NPs offer a transformative platform with significant potential for future biomedical applications by combining photothermal capabilities and biocompatibility.

## 1. Introduction

For decades, nanoparticles exhibiting localized surface plasmon resonances (LSPRs) have been tailored to various biomedical applications: multimodal diagnostic imaging, ultrasensitive analyte detection, and photothermal, photodynamic, and chemodynamic therapies [1]. Noble metals plasmonic NPs were dominating the field, with current efforts being paid to the enhanced LSPR tuning in near-infrared [2], targeted surface functionalization [3], and lowering NPs’ toxicity [4]. Recently, other materials have made their way into the NIR theranostic field, including, for example, copper chalcogenide semiconducting NPs because of their cheapness and their broadly NIR tunable LSPRs allowing remote through-tissue therapeutics via NIR transparency windows [5]. However, those particles often struggle with low biocompatibility, i.e., limited clearance pathways and high in vivo biotoxicity due to the potential release of coppery ions [6]. In contrast, the other class of semiconductor plasmonic NPs, represented by doped metal oxides (MOs), exhibits enhanced biocompatibility and flexibility in various diagnostic and therapeutic applications [7], e.g., tin oxide is already an approved and stable cosmetic additive [8].

Antimony-doped tin oxide (ATO) NPs offer a unique advantage for cancer treatment: their NIR light absorption properties can be precisely adjusted by modifying their surface chemistry in water-based solutions [9]. This tunability makes them ideal candidates for deep-tissue cancer theranostics, which combines diagnosis and therapy in Squamous Cell Carcinoma 15 (SCC15) and HeLa cancer cells [10,11]. ATO NPs are an attractive alternative to conventional plasmonic MOs like those containing tungsten and indium. This is due to their cost-effective aqueous synthesis and inherent hydrophilicity, simplifying processing and promoting biocompatibility [12]. Notably, the NIR light absorption of ATO NPs [12] aligns perfectly with the transparent windows in human tissue at 1750 nm (NIR-III) and 2200 nm (NIR-IV) wavelengths [13]. This translates to their potential for non-invasive imaging and targeted heat-based destruction of cancer cells, even in deep tissues (centimeter-scale depth) [13]. C. Gao et al. [14] introduced a competitive strategy to induce NIR LSPRs in tin oxide NPs by engineering oxygen vacancies. By reducing the commercially available tin oxide with sodium borohydride, SnO_2−x_ NPs successfully underwent plasmonic photothermal treatment (PPTT) in vivo. Although doping with oxygen vacancies made plasmonic NPs more sensitive to oxidation during ambient exposure, using aliovalent cation exchanges, as performed in this study, enhanced their chemical and thermal stability [15].

Many studies have explored the various mechanisms by which NPs enter cells [16]. However, these mechanisms are often based on gold or silica NPs [17,18], and less is known about the uptake of semiconductor plasmonic nanoparticles like ATO NPs [16]. This lack of knowledge hinders our understanding of whether photothermal tumor ablation is due to localized heating within the targeted cancer cells or a collective heating effect involving surrounding tissues. A significant challenge in nanomedicine is the low delivery efficiency of nanotherapeutic agents to tumors. Typically, less than 1% of the administered dose reaches the target; colloidally stable NPs with higher zeta potential (|ζ| > 10 mV) have further reduced uptake [19]. Understanding non-targeted uptake by macrophages before in vivo experiments is crucial to minimize potential side effects. Macrophages are sentinel immune cells throughout the body and play a key role in engulfing foreign materials, including nanoparticles [20,21]. Therefore, investigating their interaction with ATO NPs is a critical first step in understanding these nanoparticles’ potential therapeutic applications and biocompatibility.

This study focused on demonstrating mouse macrophages’ cellular uptake of ATO NPs. Visualizing their uptake serves as a proof of concept for their potential internalization by these immune cells. This information is valuable for future research on optimizing ATO NP delivery and therapeutic efficacy for cancer treatment.

## 2. Materials and Methods

### 2.1. Materials

Metallic granular tin (Sn, 99.5+%), antimony (III) oxide (Sb_2_O_3_, 99%), nitric acid (ACS grade), ethanol (ACS grade), toluene, tetrachlorethylene (TCE, ACS grade), triethylamine (TEA, (C_2_H_5_)_3_N, 99%), and oleylamine (OA, CH_3_(CH_2_)_15_(CH)_2_H_2_N, >70%) were received from Sigma-Aldrich (Merck KGaA, Darmstadt, Germany).

### 2.2. Syntheses

Antimony tin oxide (ATO) and tin oxide (TO) nanocrystals were synthesized by a modified aquathermal procedure from [12]. Six mmol of tin and 0.6 mmol (0 mmol for TO) of Sb_2_O_3_ were dissolved in 18 mL of deionized water and 11 mL of nitric acid (added dropwise to water) under vigorous stirring at room temperature. Afterward, the mixture was transferred into a 50 mL Teflon-lined stainless-steel autoclave, sealed, and the reaction ran at 180 °C for 15 h. The obtained NPs were separated by centrifugation (10 min at 8000× *g*) and washed twice with deionized water. Afterward, ca. 50 µL of TEA was added dropwise to the solution, stabilizing NPs for prolonged storage without observed flocculates. Additionally, a batch of NPs was washed sequentially with ethanol and toluene and stabilized by adding a similar quantity of oleylamine (OA). This batch was redispersed in an IR transparent organic solvent, namely TCE, to locate the position of the plasmon peak. 

### 2.3. NPs Analytical Characterization

Particle size analysis was conducted utilizing the Litesizer particle size analyzer from Anton Paar, Austria. The initial stock suspensions were further diluted using Milli-Q water to achieve nanoparticle concentrations of 25 and 100 μg/mL. The measurements were consistently carried out at a temperature of 37 °C. Data collection occurred at a fixed scattering angle of 90°, employing a Semiconductor laser diode with an output of 40 mW and a wavelength of 658 nm. The mean particle hydrodynamic size and the corresponding standard deviation were derived from three distinct measurements. The surface charge was determined through phase-amplitude light scattering, utilizing the ZetaPALS system from Brookhaven Instruments Corp., based in Holtsville, NY, USA. This analysis was conducted in a Milli-Q water environment. The NPs crystalline structure and crystallites’ sizes were estimated via XRD in the Bragg–Brentano geometry using D8 Advance X-ray powder diffractometer (CuK_α1_ radiation, Bruker AXS GmbH, Karlsruhe, Germany) in the sliding angles range 2Θ: 2–90° with the 0.025° step. UV-Vis-NIR absorbance spectra (300–2700 nm) of NPs in TCE/water/cRPMI were measured using a V-670 spectrophotometer (Jasco, Oklahoma City, OK, USA) in 10 mm path-length two-polished sides quartz cuvettes.

### 2.4. Endotoxin Content

The Endotoxin content of the NP solutions was assessed using the end-point chromogenic Limulus Amebocyte Lysate (LAL) assay. The Endotoxin content of the ATO NP suspension was evaluated at 25 µg/mL and 100 µg/mL. Solutions were then collected in sample tubes, and their endotoxin content was quantified using the Pierce Chromogenic Endotoxin Quant Kit (Cat. No.: A39552). The assay was performed according to the manufacturer’s protocol using the high-standard method. 

### 2.5. Cell Culture and ATO NP Exposure

Mouse macrophages J774A.1, purchased from the American Type Culture Collection (ATCC, Rockville, MD, USA), were cultivated in Roswell Park Memorial Institute 1640 medium (RPMI; Cat. No. 11835030, Gibco, Life Technologies, Zug, Switzerland), supplemented with 10% *v/v* fetal bovine serum (FBS), 1% *v/v* penicillin/streptomycin, and 1% *v/v* L-glutamine, referred to as complete RPMI (cRPMI). Cells were maintained in polystyrene cell culture flasks (TRP, Trasadingen, Switzerland) with a surface area of 75 cm^2^ in cRPMI media and kept at 37 °C, 5% CO_2_, and 95% relative humidity. When reaching 70–80% confluence, cells were subcultured by scraping and resuspended in 10 mL of fresh cRPMI medium. Cell viability was determined by an automated cell counter (EVE, NanoEnTek Inc., Seoul, Republic of Korea) via the erythrosine B (0.2% in PBS, *v/v*) exclusion method. Cells were grown to 70–80% confluence for seven days before seeding on a well plate. For the WST-1 assay, cells were seeded in a 24-well plate (Falcon^®^, Burlington, VT, USA) at a density of 481,000 cells/mL (50,000 cells/well). Cells were incubated at 37 °C, 5% CO_2_, and 95% relative humidity for 24 h before being exposed to Lipopolysaccharide (LPS: 0.1 μg/mL; positive control for immune reaction), Triton X-100 (positive control for cytotoxicity assay), or ATO NPs.

### 2.6. ATO NPs Photothermal Treatment in J774A.1 Mouse Macrophages

Cells were plated in µ-Slide 8 Wells (Ibidi, Graefelfing, Germany) at a density of 481,000 cells/mL (144,400 cells/well). An excitation laser of λ = 1064 nm fiber laser (YLR-10-1064-LP, IPG photonics, Oxford, MA, USA) was used to conduct the irradiation experiments. The irradiation was performed after a 24 h exposure to the ATO NPs (25 μg/mL and 100 μg/mL). Three distinct laser powers were employed (1 W/cm^2^, 5 W/cm^2^, and 10 W/cm^2^), and the irradiation was carried out for 1 min and 2.5 min for each power level. The same conditions were utilized for the corresponding negative control, i.e., cells without NPs. The cell survival rate was detected by WST-1 assay 24 h after irradiation.

### 2.7. Phase Contrast Pictures

Phase contrast pictures at a magnification ×10 were captured (Motic, AE2000 Inverted Microscope Motic Deutschland GmbH, Wetzlar, Germany) to characterize the morphology of the cells following ATO NP exposure.

### 2.8. WST-1 Assay

The WST-1 assay (WST-1, Cat. #5015944001, Roche Diagnostics, Rotkreuz, Switzerland) was used for spectrometric quantification of metabolic activity. After removing the cell culture medium, J774A.1 cells were incubated for one hour with 100 µL of freshly prepared WST-1 solution (diluted 1:10 in cRPMI) according to the manufacturer’s protocol. As a positive control, cells were exposed to 0.2% Triton X-100 (diluted in cRPMI; *v/v*) and incubated for 24 h at 37 °C, 5% CO_2_, and 95% relative humidity. Absorbance was determined at a wavelength of 440 nm (Benchmark microplate reader, BioRad, Cressier, Switzerland). The percentage of metabolic activity was quantified and expressed relative to the negative control (untreated cells), which was set as 100%. The experiment was performed with at least three biological replicates and three technical replicates for each condition.

### 2.9. ELISA: IL-6 and TNF-α

The cell supernatants of mouse macrophages J774A.1 exposed to ATO NPs (25 μg/mL), LPS (0.1 μg/mL), or negative control (untreated), were collected and stored at −80 °C for subsequent analysis. According to the supplier’s protocols, the amount of released pro-inflammatory cytokines IL-6 and TNF-α was determined using a DuoSet ELISA Development Kit (R&D Systems, Zug, Switzerland). The measurements were performed in high-binding polystyrene 96-well plates (Corning Sigma Aldrich, St. Louis, MO, USA). Standards and samples were run in three technical replicates with three biological replicates. The concentrations of IL-6 and TNF-α released in the cell culture medium were calculated based on the standard curves and fitted with a four-parameter logistic (4PL) approach using GraphPad Prism 8 software (GraphPad Software Inc., San Diego, CA, USA). Results are expressed as pg/50,000 cells of released cytokine.

### 2.10. Focused Ion Beam-Scanning Electron Microscopy (FIB-SEM) Imaging

After the NP incubation and fixation experiment, the cells were fixed with 3% glutaraldehyde and partially embedded in epoxy resin [22]. A thin layer of gold (~4 nm) was sputtered onto the sample surface to increase the conductivity of the sample. A Thermo Scientific Scios 2 DualBeam microscope (Thermo Fisher Scientific, Waltham, MA, USA) was used for FIB-SEM imaging. A platinum layer (~1 µm) was deposited on top of the region of interest to protect the area of interest during the cross-section. This SEM visualization was complemented by energy-dispersive X-ray (EDX) analysis for the characteristic X-rays (O Kα, C Kα, Ga Lα, Sb Lα, Sn Lα) to determine the elemental composition of the exposed area of interest.

### 2.11. Transmission Electron Microscopy (TEM)

To examine the morphology of ATO NPs, a Tecnai Spirit transmission electron microscope (TEM; FEI Technai G2 Spirit, Thermo Fisher Scientific, Waltham, MA, USA) equipped with a Veleta CDD camera (Veleta, Olympus, Tokyo, Japan) and operating at 120 kV was used. NPs were centrifuged at 16,000× *g* for 30 min and redispersed in Milli-Q water. The resuspended NPs were then drop-cast onto 300-mesh Formvar copper grids to visualize the pristine ATO NPs. 

To observe the intracellular uptake of ATO NPs, J774A.1 cells were incubated with complete culture media containing 25 μg/mL and 100 μg/mL at 37 °C for 24 h. All cell samples were fixed with aldehyde solutions, post-fixed with osmium tetroxide and stained with lead aspartate solution, dehydrated with a series of ethanol solutions, and gradually infiltrated with Epon epoxy resin, which was allowed to polymerize at 60 °C for 48 h. Then, the samples were sectioned with a diamond knife mounted on an ultramicrotome. The ultrathin sections were supported on a copper grid. Finally, TEM images were analyzed using ImageJ 1.51 software (National Institutes of Health, Bethesda, MD, USA).

### 2.12. Lock-In Thermography (LIT)

LIT measurements were performed using a custom-made setup with an infrared camera (Onca-MWIR-InSb-320, XenICs, Leuven, Belgium) operating at a frame rate of 200 Hz. The modulation LED frequency and the number of cycles during acquisition was set to ½ Hz and 50, respectively. We adjusted the electric power to 1 W for each LED during each measurement. The samples were measured in a dried state by drop-casting the different ATO NP solutions on a glass cover. 

J774A.1 macrophages were seeded and allowed to adhere to culture plates. To investigate cellular uptake, the cells were exposed to ATO NPs at two concentrations (25 μg/mL and 100 μg/mL) for 24 h. Following incubation, the cells were fixed, dried, and prepared for analysis of both cellular uptake and photothermal properties. 

### 2.13. Statistical Analysis

Experiments were performed in at least three biological replicates and duplicate or triplicate technical replicates. The data shown are average values ± S.D. Statistical analysis was performed by one-way ANOVA followed by Dunnett’s multiple comparisons test with a single pooled variance (GraphPad Software, Inc.), assuming equal variances with *p* < 0.05, * *p* < 0.05, ** *p* < 0.01, *** *p* < 0.005, and **** *p* < 0.001.

## 3. Results

ATO NPs were prepared by the feasible surfactant-free aquathermal reaction between metallic tin and antimony (III) oxide (molar ratio: 1.0/0.1) in diluted nitric acid. This procedure is beneficial primarily due to the one-step approach, not requiring organic solvents/add-ons which tend to leave a residue on the NP surface even after washing [11]. Since prepared nanoparticles have poor hydrophilicity due to their zeta potential being in the proximity of the isoelectric point, thus the short-chain amine molecules [9] are employed to produce scalable and long-term NP dispersibility in aqueous solutions. The used antimony feed ratio is optimal to preserve the highest NP optical density in the NIR region [12] via the specific mechanism for Sb-concentration quenching in the tin dioxide lattice. The latter is drawn as a stepwise aliovalent substitution of Sn^4+^ ions at lower Sb concentrations by ns^0^ ions Sb^5+^, generating electrons to the NP conduction band. This process quenches at higher antimony concentration, wherein acceptor-type ns^2^ Sb^3+^ ion doping dominates, resulting in free carriers trapping. 

**Figure 1:**
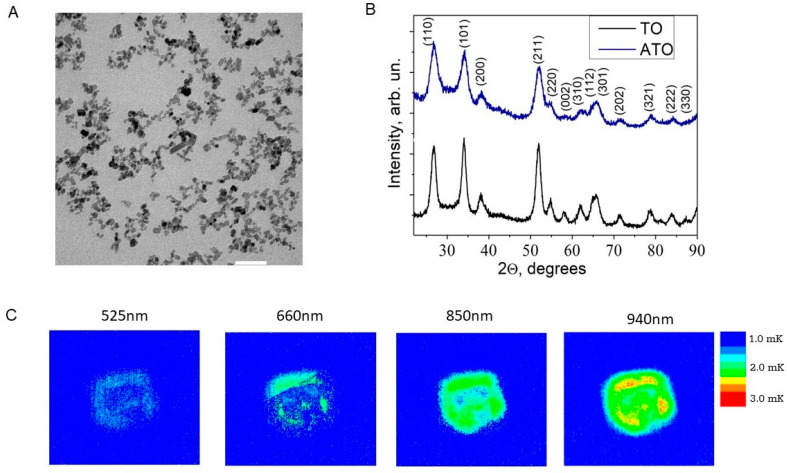
Characterization of ATO NPs. (**A**): Representative TEM image of ATO NPs Scale bar: 50 nm; (**B**): XRD patterns of ATO and TO NPs; (**C**): LIT thermal maps of pristine ATO NPs, spin-casted at 5000 rpm for 30 s on a glass coverslip, subjected to a series of diverse monochrome LED excitations.

The core particle diameter was initially measured by transmission electron microscopy. The ATO NPs have a slightly elongated rectangular shape with a mean diameter of about 5 nm (Figure 1A). The NPs’ hydrodynamic diameter was assessed in Milli-Q water and complete cell culture medium using dynamic light scattering (DLS) at two different concentrations of ATO NPs (25 μg/mL and 100 μg/mL). The NPs form agglomerates, observed after drying on the TEM grid and hydrodynamically with DLS (Table S1). The zeta potential was determined at −32.3 mV (Table S1) and negligibly altered within the concentration ranges studied. This is consistent with the theory of dissociated short-chain amines [9] forming a strong negative diffuse layer within the notional boundary of the ATO NP surrounding. The crystalline structure of ATO NPs inherits the structure of undoped tin dioxide (cassiterite, JCPDS 41-1445, see Figure 1B and Table S2) with lattice parameters slightly altered with antimony doping. The diffraction reflexes in the TO/ATO samples perfectly match the specific d_hkl_ distances for the orthorhombic cassiterite crystal structure (Figure 1B, Table S2). For the ATO sample, simultaneous peaks’ shifts (Figure 1B) toward higher diffraction angles indicate a lattice contraction (Table S2), evidencing the antimony incorporation into the lattice predominantly by smaller (than host Sn^4+^) donor-type Sb^5+^ ions. Crystallite sizes calculated by the Scherrer equation roughly correspond to those deducted by TEM (Figure 1A, Table S2), assuming minor NP aggregation during drying.

ATO materials start manifesting the resonant interaction with the light right after the bandgap offset due to the excitation of oscillation of free electrons provided to the valence band by aliovalent (Sb in the Sb^5+^ ns^0^ state) dopant. Thus, optical absorbance has been collected in UV-Vis-NIR of the NPs dispersed in water (Figure S1A) (up to 1500 nm) and TCE (Figure S1B) (up to 2600 nm). An LSPR peak is observed at a wavelength of ca. 2300 nm for the ATO sample, matching thus perfectly with an NIR-IV tissue transparency window. The optical absorbance drops ca. twice and ca. four times for the regions attributed to NIR-III and NIR-II windows. Still, their absolute values harness ATO NPs as promising photothermal tools when excited by shorter NIR electromagnetic waves. 

The excellent photothermal properties of ATO NPs under NIR light irradiation are a significant advantage that is absent in pristine TO, as shown in Figure S1C. Upon exposure to light, the collective oscillation of excited electrons within the nanoparticles translates into heat through localized or collective heating effects. As shown in Figure 1C, ATO NPs are efficient photothermal heaters across a broad range of visible and near-infrared wavelengths. This tunability offers excellent potential for diverse applications. The observed thermal profiles generated by the nanoparticles closely mirror their absorbance patterns. As expected, increasing the excitation wavelength (with constant LED power) towards the peak of the NIR LSPR band resulted in a progressively stronger thermal gradient on the maps. This finding, evaluated by LIT mapping, provides strong evidence for the plasmonic nature of the NIR absorbance and underscores the potential efficacy of ATO NPs for photothermal therapy in the NIR region.

### 3.1. Interaction of ATO NPs with J774A.1 Macrophages

**Figure 2:**
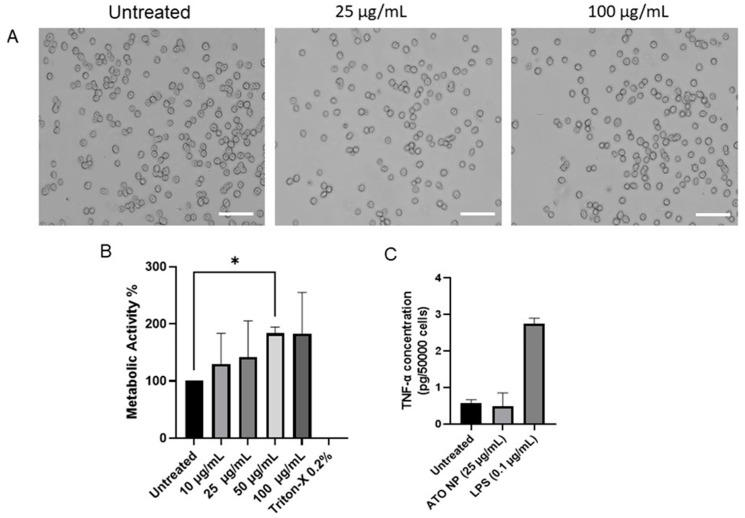
Cellular morphology, viability assessment, and cytokine release. (**A**): Representative phase-contrast images of J774A.1 cells upon exposure to ATO NPs for 24 h at 25 μg/mL and 100 μg/mL; (**B**): Cell viability was evaluated in J774A.1 macrophages using the WST-1 assay after exposure for 24 h to ATO NPs; (**C**): Cytokine secretion was assessed in J774A.1 to ATO NPs (25 µg/mL) for 24 h using TNF-α ELISA. Cells were exposed to 0.1 μg/mL LPS as a positive control. Data shown are mean values ± S.D. (n = 3). Scale bars: 100 µm. Data are shown as means ± SD. n = 3. The statistical analysis was conducted using one-way ANOVA followed by Dunnett’s multiple comparisons test with a single pooled variance, *p* < 0.05 (*).

We employed the mouse macrophage J774A.1 cell line as our model to evaluate cell morphology and viability. Before using the ATO NPs, we determined their endotoxin levels using the Limulus amebocyte lysate (LAL) assay, and the values were found to be 0.1–0.3 EU/mL for ATO NPs at 25 μg/mL and 100 μg/mL, as shown in Supplementary Table S3. This step is crucial as nanomaterials can be contaminated with bacterial endotoxin during production, potentially confounding the interpretation of toxicological tests, especially those investigating immunotoxicity [21,23]. Phase-contrast imaging was then employed to examine possible changes in cell morphology after exposure to ATO NPs for 24 h in J774A.1 macrophages. Our results, as shown in Figure 2A, demonstrate that the morphology of J774A.1 macrophages remained like the untreated control after exposure to ATO NPs (25 μg/mL and 100 μg/mL). 

Also, the WST-1 assay (Figure 2B) demonstrated no significant reduction in cell viability of J774A.1 macrophages exposed to ATO NPs (up to 100 μg/mL) for 24 h compared to the untreated control. This assay assesses mitochondrial dehydrogenase activity, which can be directly associated with the number of viable cells [24]. Further, the WST-1 assay revealed a significant increase in metabolic activity at 50 μg/mL of ATO NPs compared to the control (Figure 2B). This suggests that J774A.1 macrophages alter their metabolic activity upon association with ATO NPs.

To further evaluate the impact of the ATO NPs on macrophage polarization, we performed an ELISA on pro-inflammatory cytokines IL-6 and TNF-α. While M1 macrophages do not exclusively produce these cytokines, they can be indicative of a proinflammatory response [25]. J774A.1 macrophages were exposed to ATO NPs (25 µg/mL) for 24 h, and LPS (0.1 µg/mL) was used as a positive control for M1 polarization. Likewise, we did not observe any secretion of IL-6; the values were below the detection limit after exposure to ATO NPs. In contrast, the effect of TNF-α, as shown in Figure 2C, was negligible compared to the untreated control. However, LPS triggered TNF-α and IL-6 release in the J774A.1 macrophages, as expected for a positive control, inducing a pro-inflammatory response. 

### 3.2. Cellular Uptake and Photothermal Response of ATO NPs

**Figure 3:**
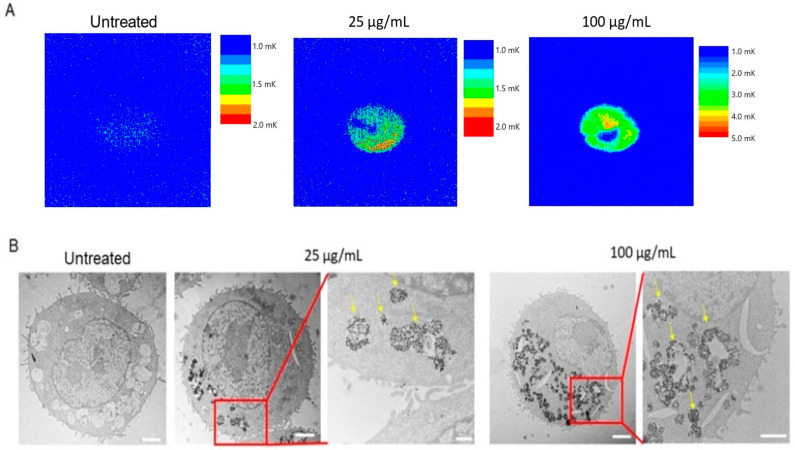
Cellular uptake of ATO NPs. (**A**): LIT thermal maps, collected upon irradiation with a 940 nm LED source upon exposure to J774A.1 cells for 24 h at 25 μg/mL and 100 μg/mL; (**B**): TEM micrographs of J774A.1 cells untreated and exposed for 24 h to ATO NPs (25 μg/mL and 100 μg/mL). The areas in the red boxes were examined at 25 μg/mL and 100 μg/mL to visualize the uptake of ATO NPs. Yellow arrows indicate the ATO NPs inside the vesicles of J774A.1 cells at these concentrations. Scale bars: 2 µm (500 nm on the magnified selected areas).

**Figure 4:**
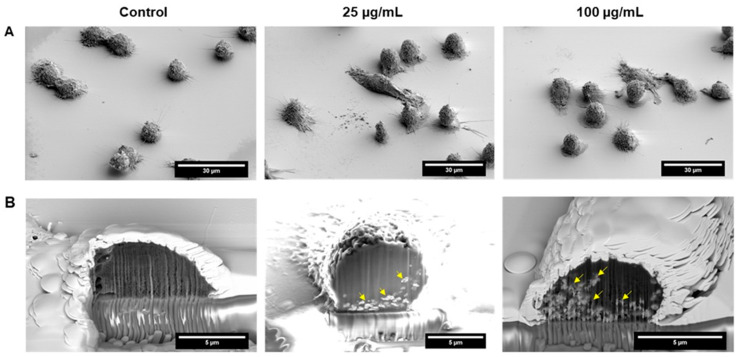
Imaging cell-ATO nanoparticles interaction by FIB-SEM. (**A**): SEM image of J774A.1 exposed to ATO nanoparticles (overview) Scale bars: 30 µm indicated. (**B**): SEM image of J774A.1 cross-section after FIB milling reveals internalized ATO nanoparticles (yellow arrows) Scale bars: 5 µm indicated.

The photothermal properties of internalized ATO NPs within J774A.1 macrophages were assessed using LIT imaging. LIT thermal maps (Figure 3A) illustrate the temperature distribution within macrophages following NIR light irradiation at 940 nm. A clear correlation between NP concentration and heating efficiency was observed, as shown in Figure 3A. Macrophages exposed to a higher concentration of ATO NPs (100 μg/mL) exhibited a stronger heating response than those incubated with a lower concentration (25 μg/mL). This observation aligns perfectly with the enhanced cellular uptake at higher NP concentrations, as demonstrated by TEM and FIB-SEM analyses (Figure 3B and Figure 4B). 

Further, to understand the internalization of ATO NPs after a 24 h incubation, the sections cells were analyzed by TEM, which revealed the efficient internalization of ATO NPs by J774A.1 macrophages (Figure 3B). Compared to the untreated control, macrophages exposed to ATO NPs displayed numerous dark spots within their intracellular compartments, confirming the presence of internalized ATO NPs. Magnified TEM images (Figure 3B) show these dark spots corresponding to ATO NPs localized within intracellular vacuoles (indicated by yellow arrows). This observation suggests that the primary uptake pathway involves endocytosis, where NPs are engulfed by the cell membrane and transported into vacuoles. 

### 3.3. Cellular Uptake and Elemental Analysis of ATO NPs in Macrophages

SEM subsequently probes FIB-prepared cell cross-sections to validate the uptake mechanism further. As shown in Figure 4A, the SEM image presents an overview of a control J774A.1 macrophage and those exposed to ATO nanoparticles. Those ATO NPs are indicated by the yellow arrows in representative SEM images (Figure 3B) collected after the FIB sectioning of a J774A.1 macrophage following incubation with ATO NPs at 25 µg/mL and 100 µg/mL concentrations. As expected, we see an increase in the uptake of NPs as the concentration increases to 100 µg/mL, which can be attributed to the higher number of ATO NPs available for clearance cell uptake. In addition to the uptake data, some elongated cells were observed in the FIB-SEM images of macrophages exposed to ATO NPs, which might suggest morphological changes or cell activation responses in the presence of the particles. This elongation could indicate cytoskeletal rearrangements or activation states often associated with phagocytosis and other immune responses. Macrophages can change shape as part of their activation process, potentially to enhance their ability to engulf and process foreign materials such as nanoparticles [26].

EDX analysis on a cell cross-section at four distinct FIB-SEM locations revealed the chemical fingerprint of the ATO NPs (25 µg/mL) following uptake, as shown in Figure S2. EDX spectra (Figure S2 (points 3–4)) show the tin (Sn) and antimony (Sb) signals, which confirm the presence of ATO NPs. That is also evidence of the stability of ATO NPs, as EDX signals from antimony and tin are always coupled. In the case of a metal ion solvation, one would expect the signals decoupling and, if both metals are dissolved, homogenous distribution of Sn and Sb throughout the entire examined cell, which was not observed. That was predictable as selected ATO materials can withstand high temperatures in solar concentrators [27] and corrosive media [28]. The light elements, namely carbon (C) and oxygen (O), were detected within the whole cross-section due to the organic nature of the pristine cell material, as shown in Figure S2 (points 1–4). Examining the O Kα and C Kα X-ray intensities, one spots the increase of oxygen-to-carbon ratio (Figure S2B) within the areas where Sn and Sb are co-detected. The source of additional oxygen present is also attributed to ATO NPs. As it is well accepted, gallium (Ga) traces were also observed due to this cation implantation into the specimen during the ion milling interaction.

### 3.4. In Vitro Photothermal Therapy Using ATO NPs

**Figure 5:**
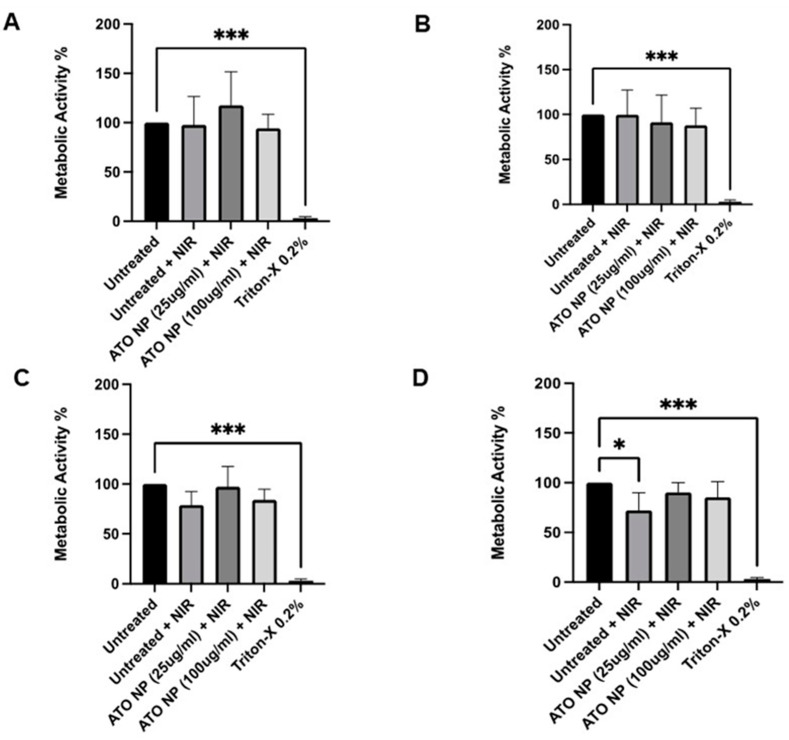
Cell viability of J774A.1 macrophages 24 h after the NIR laser (1064 nm) irradiation using the WST-1 assay. (**A**): Laser power 1 W/cm^2^, time of irradiation 1 min; (**B**): Laser power 1 W/cm^2^, time of irradiation 2.5 min; (**C**): Laser power 5 W/cm^2^, time of irradiation 1 min; (**D**): Laser power 5 W/cm^2^, time of irradiation 2.5 min. Data are shown as means ± SD. n=3. The statistical analysis was conducted using one-way ANOVA followed by Dunnett’s multiple comparisons test with a single pooled variance, *p* < 0.05 (*) and *p* < 0.001 (***) compared to Untreated.

Given the high photothermal conversion efficiency exhibited by the synthesized ATO NPs, these ATO NPs offer substantial potential as exceptional agents for photothermal therapy. We further investigated the photothermal effect of ATO NPs internalized by the cells by assessing cell viability after NIR laser irradiation at different power densities and irradiation times. J774A.1 macrophages were cultured with 25 μg/mL ATO NPs for 24 h before exposure to NIR laser irradiation (1064 nm). Cell viability was then measured using the WST-1 assay (Figure 5). Interestingly, the data in Figure 5A–D show no significant changes in metabolic activity compared to the untreated control upon irradiation with increasing laser power densities (1 W/cm^2^, 5 W/cm^2^) and longer irradiation times (1 min, 2.5 min). This suggests that under these conditions, the combination of ATO NPs and NIR laser irradiation may not be sufficient to induce substantial cytotoxicity in macrophages. 

However, at a higher laser power density of 10 W/cm^2^ (Figure S3A,B), a significant decrease in cell viability was observed even with a short irradiation time (1 min). These findings warrant further investigation to optimize the laser parameters (wavelength, power density, and irradiation time) for achieving a controlled and effective photothermal response in macrophages with minimal damage to healthy cells.

## 4. Discussion

ATO NPs exhibit promising potential as innovative platforms for developing novel anticancer NIR photothermal agents owing to their distinctive defect structure [10,29]. They combine tunable photothermal conversion matching NIR-II–NIR-IV tissue transparency windows with preserved stability and LSPR response in the cell culture media. Our study sheds light on the intriguing interaction between ATO NPs and macrophages, highlighting the critical role of macrophage biology in developing ATO NPs for cancer therapy. The unique properties of ATO NPs, including their tunable photothermal conversion efficiency and excellent biocompatibility (Figure 2), make them promising candidates for next-generation PTT agents. However, their therapeutic efficacy hinges on their direct cytotoxic effects on cancer cells and their ability to modulate the tumor microenvironment.

The consistent reproducibility of the synthesis process, coupled with minimal batch-to-batch variability and lower endotoxin levels (as shown in Table S3), underscores the potential of ATO NPs to meet the rigorous standards mandated for pharmaceutical products and medical devices [23,30]. These findings indicate that ATO NPs exhibit numerous favorable attributes, notably colloidal stability within pertinent environments, as shown in Table S1. Nonetheless, it is essential to acknowledge that developing a protein corona within living organisms might impact the NP’s pharmacokinetics. Consequently, investigating this aspect further in animal models becomes an imperative avenue for exploration [31].

Macrophages play a central role in the innate immune system and orchestrate complex immune responses within the tumor microenvironment [32]. Their remarkable plasticity allows them to adopt different phenotypes, ranging from pro-inflammatory M1 to anti-inflammatory M2 macrophages [33]. Notably, tumor-associated macrophages (TAMs) are a heterogeneous population within tumors, often exhibiting an immunosuppressive M2 phenotype that promotes tumor growth and metastasis [34]. Our findings demonstrate the efficient internalization of ATO NPs by J774A.1 macrophages, even without specific targeting agents (Figure 3B and Figure 4B). By strategically encapsulating therapeutic agents within ATO NPs, we can potentially deliver them directly to macrophages within the tumor microenvironment, maximizing therapeutic efficacy and minimizing systemic exposure. Understanding this intricate interplay between ATO NPs and macrophages is crucial for optimizing their therapeutic potential. By manipulating macrophage behavior towards a more pro-inflammatory M1 phenotype, ATO NPs could directly target cancer cells via PTT and stimulate the immune system to combat tumor progression.

Notably, the observed changes in macrophage behavior—such as increased uptake of ATO NPs and potential shifts in activation states [35,36]—suggest that these nanoparticles might be leveraged to enhance the therapeutic efficacy of macrophages in targeting cancer cells. For example, ATO NPs could induce macrophages to adopt a more pro-inflammatory M1 phenotype [37], known to have anti-tumor effects [38]. This shift could potentially improve the effectiveness of photothermal therapy by targeting cancer cells directly and enhancing the overall immune response within the tumor microenvironment [39]. Further research is needed to explore how these macrophage changes translate into improved anti-cancer effects, including in vivo studies that assess the interaction between ATO NPs, macrophages, and tumor cells [40].

However, addressing the observed discrepancies in photothermal responses is vital when different light sources and power densities are employed. The decent thermal response differences between J774A.1 cells exposed for 24 h at 25 μg/mL and 100 μg/mL of ATO NPs under 940 nm irradiation (Figure 3A) does not appear as pronounced when a 1064 nm laser at 10 W/cm^2^ is used (Figure S3A,B). This discrepancy can be attributed to the substantial readout differences for the LIT and PPTT probes. The former is an ultrasensitive modulation technique capable of resolving ultrasmall thermal gradients, which serves as additional evidence of the ATO NP uptake. The PPTT generates less-pronounced concentration dependencies in thermal effect as it may solely overcome the cells’ hyperthermia level, which is seen for the ATO untreated sample. The nonlinear effects in heat dissipation at high power density may further contribute to the observed differences, underscoring the importance of optimizing PPTT parameters, namely laser wavelength and power, duration of treatment, and the precise control of initial temperature [41] for maximizing the photothermal efficiency of ATO NPs for the targeted cells. 

Biocompatibility is a critical issue in nanomedicine. In this study, neither activated nor non-activated ATO nanoparticles (NPs) caused a substantial decrease in the metabolic activity exhibited by J774A.1 cells at concentrations up to 100 μg/mL. This observation aligns with the absence of any noticeable abnormal morphological alterations, as indicated by FIB sectioning, TEM micrographs, and phase contrast images. The notable maintenance of cell viability following NIR irradiation up to 5 W/cm^2^ serves to confirm the suitability of ATO NPs as biocompatible agents for PPTT, which aligns with the previous reports on macrophages and neutrophils for various classes of nanomaterials [42,43]. Nonetheless, a thorough investigation is required into primary macrophages or neutrophils, which constitute the pivotal elements of the innate immune system [44]. In particular, the degradation of NPs by macrophages or neutrophils augments their biocompatible characteristics and provides insights into potential clearance mechanisms [45].

In response to extracellular signals, macrophages undergo phenotypic changes through a cytokine secretion process that holds significant importance in the context of tumor-infiltrating immune cells [33]. Also, there have been recent reports on carbon-based materials in which there is a modulation of IL-10 and TNF-α secretion [42,46]. Nonetheless, in our investigation, ATO NPs did not trigger the secretion of either IL-6 or TNF-α, even without cellular death. Undoubtedly, tumor-associated macrophages are pivotal controllers of the immune reaction against solid tumors. The nuanced interplay between ATO NPs and macrophages adds a layer of complexity to the therapeutic landscape, warranting meticulous investigation of relevant animal models. The fusion of ATO NPs’ photothermal properties, biocompatibility, and the intriguing modulation of macrophage responses unveil an avenue for tailored and potent anticancer interventions [34].

While our in vitro studies provide valuable insights, evaluating the biodistribution of ATO NPs within the body and their interaction with primary macrophages will be crucial for translating these findings into clinical applications. Additionally, exploring the therapeutic efficacy of ATO NP-mediated PPTT in combination with macrophage immunomodulation, in vivo cancer models will be paramount in establishing ATO NPs as a transformative theranostic platform for cancer treatment. Optimizing the particle size, surface coating, and concentration will be necessary in maximizing their photothermal effect and ensuring safe and effective delivery to target tissues. Demonstrating the phototoxic effect of ATO NPs in vitro is a crucial step before advancing to in vivo studies, as it will confirm their potential to induce localized cell death upon NIR irradiation.

## 5. Conclusions

Summarizing, we investigated ATO NPs as a potential photothermal tool in anticancer therapies. The desirable features of ATO NPs, such as their wavelength tunable photothermal conversion, colloidal stability in cell culture media, and biocompatibility, are matched with desirable macrophage responses and uptake mechanisms. However, further research is essential to assess their interaction with other immune components and behavior in more complex biological systems, mainly through in vivo studies. While our in vitro data, including the absence of significant immune reactions and detailed uptake mapping via FIB-SEM, provide valuable insights, these results are preliminary. They lay the groundwork for more comprehensive in vivo research to advance ATO NPs toward clinical translation as targeted and effective anticancer agents.

## Data Availability

The corresponding authors will provide data supporting the plots and mappings in this paper and other studies’ findings upon reasonable request.

## Supplementary Materials

The following supporting information can be downloaded at: https://www.mdpi.com/article/10.3390/cells13171468/s1, Table S1: DLS data of ATO NPs; Table S2: XRD data of TO/ATO NPs; Table S3: Endotoxin levels of ATO NPs; Figure S1: UV-Vis-NIR absorbance spectra and LIT maps of TO/ATO NPs; Figure S2: FIB-SEM cross-section with EDX probes of a cell-ATO NPs internalization; Figure S3: Cells’ viability under NIR.
