# Supplementary material for: Understanding Macrophage Interaction with Antimony-Doped Tin Oxide Plasmonic Nanoparticles
Source: Cells. 2024 Sep 1;13(17):1468. doi: 10.3390/cells13171468 (PMC11394000; doi:10.3390/cells13171468)
Supplement: Supplementary file 1 [file cells-13-01468-s001.zip › cells-3152618-supplementary.pdf]

cells

MDPI

Supplementary materials

Understanding Macrophage Interaction with Antimony-Doped Tin Oxide Plasmonic Nanoparticles

Olexiy Balitskii [1,2,*], Viktoriya Ivasiv [1,3], Maria Porteiro Figueiras [1], Phattadon Yajan [1], Mira Witzig [1], Aura Maria Moreno-Echeverri [1], José Muñetón Díaz [4], Barbara Rothen-Rutishauser [1], Alke Petri-Fink [1,5] and Sandeep Keshavan [1,*]

[1] Adolphe Merkle Institute, University of Fribourg, Chemin des Verdiers 4, 1700 Fribourg, Switzerland; viktoriya.ivasiv@unifr.ch (V.I.); maria.porteirofigueiras@unifr.ch (M.P.F.); phattadon.yajan@unifr.ch (P.Y.); mira.witzig@unifr.ch (M.W.); auramaria.morenoecheverri@unifr.ch (A.M.M.-E.); barbara.rothen@unifr.ch (B.R.-R.); alke.fink@unifr.ch (A.P.-F.)
[2] Department of Chemistry, University of Waterloo, 200 University Avenue West, Waterloo, ON N2L 3G1, Canada
[3] CQUM-Centre of Chemistry, Chemistry Department, University of Minho, R. da Universidade, 4710-057 Braga, Portugal
[4] Department of Physics, University of Fribourg, 1700 Fribourg, Switzerland; jose.munetondiaz@unifr.ch
[5] Department of Chemistry, University of Fribourg, Chemin du Musée 9, 1700 Fribourg, Switzerland
* Correspondence: olexiy.balitskii@uwaterloo.ca (O.B.); sandeep.keshavan@unifr.ch (S.K.)

Table S1. DLS measured Zeta potential and hydrodynamic diameter distributions of ATO NPs in Milli-Q water.

ATO NP concentration, μg/mL	D_h, nm	PDI	ζ. meV
25	46± 2	0.184	-31.0±1.3
100	44± 1	0.141	-32.3±1.9

Table S2. XRD data (lattice parameters and crystallite sizes variation) of the ATO (TO) NCs.

	TO (0% Sb)	ATO (20% Sb)
Lattice parameters<br>JCPDS #41-1445 for cassiterite<br>deducted from Bragg Eq.<br>$\sqrt{\frac{\boldsymbol{a^2}}{\boldsymbol{h^2+k^2}}+\frac{\boldsymbol{c^2}}{\boldsymbol{l^2}}}=\boldsymbol{d_{hkl}}=\frac{\lambda}{\boldsymbol{2\sin(\theta)}}$		
a, nm	0.4695	0.4678
c, nm	0.3201	0.3186
Crystallite sizes,<br>Deducted for (110) from Scherrer Eq.<br>$\boldsymbol{D}=\frac{\boldsymbol{0.9}\,\lambda}{\Delta(\boldsymbol{2\theta})\boldsymbol{\cos(\theta)}}$, nm	11.44	8.08

Table S3. Endotoxin levels of ATO NPs using LAL assay.

ATO NP concentration, µg/mL	EL, EU/mL
25	0.132
100	0.300

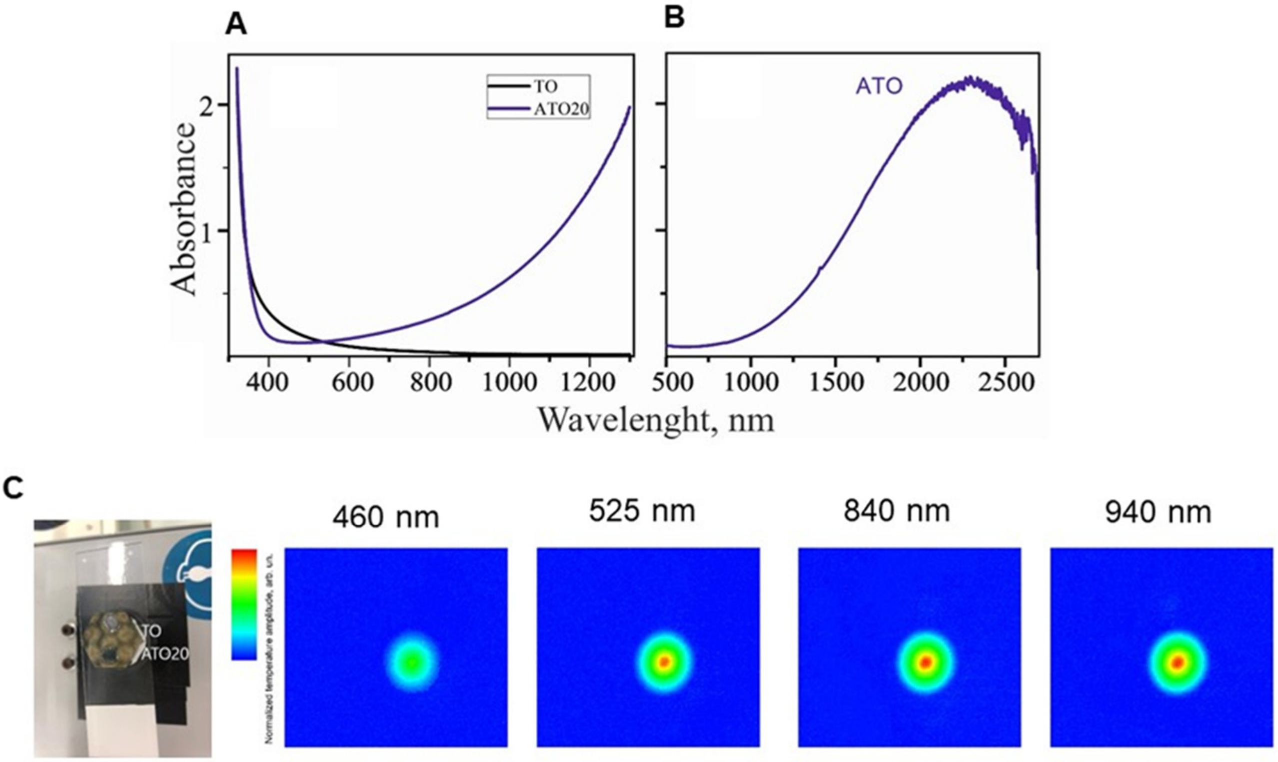

Figure S1. UV-vis-NIR absorbance spectra of (A): TO/ATO NP solutions (10 mg/mL) in water and (B): ATO NP solution (0.2 mg/mL) in TCE; (C): LIT thermal maps of TO/ATO NPs, drop-casted on a glass cover (see left-hand side photo) under diverse monochrome power-normalized LED excitations.

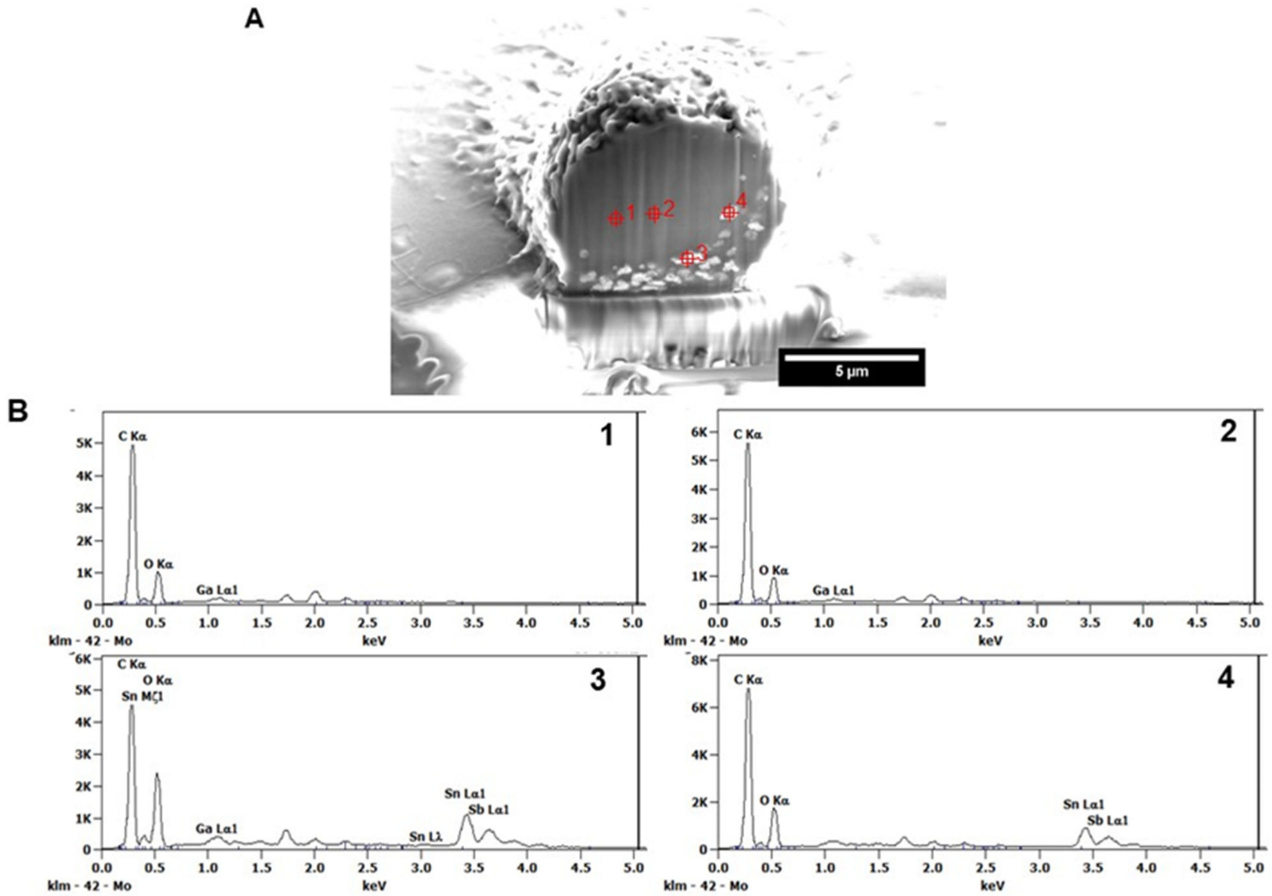

Figure S2. (A): FIB-SEM cross-section with locations of EDX probes of a cell-ATO nanoparticles (25 µg/mL) internalization; (B): Elemental probes confirm the absence (points 1 and 2) and the presence (points 3 and 4) of internalized ATO nanoparticles at the specific locations inside the cell.

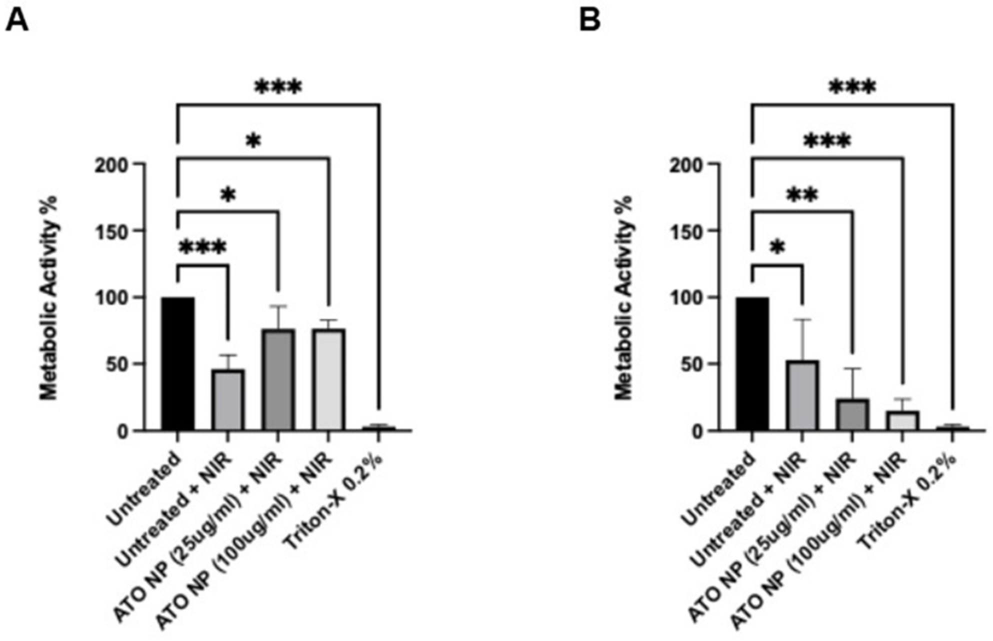

Figure S3. Cell viability of J774A.1 macrophages 24 h after the NIR laser (1064 nm) irradiation using the WST-1 assay. (A): Laser power 10 W/cm^2, time of irradiation 1 min; (B): Laser power 10 W/cm^2, time of irradiation 2.5 min; Data are shown as means ± SD. n=3. The statistical analysis was conducted using one-way ANOVA followed by Dunnett's multiple comparisons test with a single pooled variance, $p<0.05$ (*) and $p<0.001$ (***) compared to Untreated.
